# Associations between Physical Activity and Mental Health in Iranian Adolescents during the COVID-19 Pandemic: An Accelerometer-Based Study

**DOI:** 10.3390/children8111022

**Published:** 2021-11-07

**Authors:** Saeed Ghorbani, Mostafa Afshari, Melanie Eckelt, Amir Dana, Andreas Bund

**Affiliations:** 1 Department of Physical Education and Sport Sciences, Aliabad Katoul Branch, Islamic Azad University, Aliabad Katoul 4941793451, Iran; 2 Department of Sport Management, Sport Management Research Center, Sport Sciences Research Institute, Tehran 1587958711, Iran; m.afshari@ssrc.ac.ir; 3 Department of Education and Social Work, University of Luxembourg, Campus Belval, 11, Porte desSciences, Esch-sur-Alzette, L-4366 Luxembourg, Luxembourg; melanie.eckelt@uni.lu (M.E.); andreas.bund@uni.lu (A.B.); 4 Department of Physical Education and Sport Sciences, North Tehran Branch, Islamic Azad University, Tehran 1651153311, Iran; a.dana@iau-tnb.ac.ir

**Keywords:** COVID-19, physical activity, mental health, accelerometer, adolescents

## Abstract

Using self-reported questionnaires, several studies found that social isolation during the COVID-19 pandemic has significantly changed the level of physical activity (PA) in children and adolescents. Since the objectivity of self-reported PA is limited in several ways, we used modern accelerometers in this study to assess the PA levels of male and female adolescents during the pandemic-related lockdown. Moreover, the association of PA with mental health of the adolescents were analyzed. A total of 136 students (76 girls, mean age of 16.28 ± 0.97 years) from various schools in Iran wore the accelerometer (ActiGraph GT3X-BT) for seven consecutive days. Mental health was measured through the Depression, Anxiety, Stress Scale-21 of Lovibond and Lovibond (1995). Descriptive statistics and independent *t*-tests were used to analyze the PA levels of male and female students, and linear regressions were computed to examine whether PA predicts mental health status. On average, the daily time spent in moderate-to-vigorous PA (MVPA) was 18.47 min, which is clearly below the WHO guideline of at least 60 min of MVPA per day. Only 4% of adolescents showed MVPA for more than 30 min per day. Male students were significantly more active than their female peers (*p* = 0.015). As expected, MVPA was negatively associated with mental health issues such as depression, anxiety, and stress. However, the majority of adolescents reported symptoms of mild-to-moderate mental health disorders. These results emphasize the need for targeted strategies and offerings geared to young people’s needs and preferences to promote an adequate level of PA and good mental health during and after the ongoing pandemic.

## 1. Introduction

Physical activity (PA) is defined as any movement of the body produced by skeletal muscles that leads to the energy expenditure and can be executed as part of sport activities, working activities, active transportation, household activities, and recreational activities [1,2]. Research has shown that regular PA is related to numerous health benefits, for example improved cardiorespiratory and muscular fitness, strong bones, enhanced memory function and cognitive control, and reduction of depressive symptoms and obesity [3,4,5,6]. Furthermore, it has been demonstrated that PA at school age can influence the level of PA during adulthood and the public health in general [7,8]. The World Health Organization (WHO) therefore recommends that children and adolescents aged 6 to 17 years should undergo moderate-to-vigorous physically activity (MVPA) for at least 60 min per day, across the week. Conversely, it is also suggested that the amount of time spent being sedentary, particularly the amount of screen time, is limited to an appropriate level [9].

COVID-19 is the name of an infectious respiratory disease caused by a new coronavirus, which was first identified in December 2019 in Wuhan (China). The disease has spread rapidly worldwide since early 2020 [10] and affected the life of millions of people; up to now, more than 4 million of people have been killed worldwide. In order to contain the spread of the virus, the governments of almost all countries have adopted severe restrictions such as home-confinement, quarantine and social isolation [11]. Although these measures have reduced the spread of the coronavirus, they also have numerous undesirable effects on people’s lifestyles. For example, following quarantine, schools were closed and teachers used digital and/or remote methods of teaching and distance learning, so that the students (i.e., children and adolescents) spent most of their time at home, often lying down or sitting. Moreover, they had no or just limited access to PA in school and sport clubs such as physical education, school excursions, workouts and competitions. It is plausible to assume that this situation has led to a further increase in sedentary behavior in children and adolescents, which would be particularly worrying since children and adolescents did not reach the WHO guideline in pre-COVID-19 times, even under normal living conditions [12,13].

Indeed, several studies have found that the COVID-19 restrictions significantly changed the PA-level of children and adolescents [14,15,16,17]. A current review of Yomoda and Kurita [14] yields that the PA of children and adolescents generally decreased during the COVID-19 pandemic. Boys, older children and adolescents are more affected by this effect than girls and younger children, while children who live in detached houses, houses with more space, rural areas, and with more family members are less affected by this effect [14]. In the U.S, parents of 5–13-year-old children reported decreases in PA as well as increases in sedentary behaviors from the pre- to early-COVID-19 periods. Moreover, the most common types of PA during the first months of the pandemic were free play, unstructured activities (e.g., running around, tag) and going for a walk. Children were more often active at home or in the neighborhood [15]. In Europe, an observational study in ten countries indicated that approximately two-thirds of children aged 6–18 followed a structured daily schedule, and more than half were physically active during online physical education. However, only one in five of these children met the WHO recommendation [16]. Another study regarding European and Latin American countries showed a drop in PA at the beginning of the pandemic, particularly in the young people from Latin America [17].

Most studies on PA behavior during the COVID-19 pandemic are based on different self-report questionnaires [15,16,17]. While this approach is simple and economical, the accuracy and reliability of the measurement is limited due to the fact that reports on one’s own activities are often incomplete and influenced by social desirability [18]. The former is even more the case in children and youth as their everyday lives are less regular than those of adults [19]. Studies comparing PA data assessed by questionnaires and accelerometers show that people usually tend to overestimate their PA, that is to say they report more PA in a questionnaire than is recorded by an accelerometer [20,21]. Therefore, the use of accelerometer (or other objective methods such as indirect calorimetry) is currently the better way to accurately collect PA data.

In addition to the detrimental effects on PA, it has been found that social isolation as well as other changes in the everyday life of children and adolescents are associated with mental health disorders such as depression, anxiety, and stress [22,23,24,25]. For example, De Miranda et al. [23] reviewed 51 studies and identified high rates of depression, anxiety, and post-traumatic symptoms among children during the pandemic. On the other hand, there is considerable evidence that PA is positively related to numerous mental health variables [26,27,28,29]. Ahn and Fedewa [26] showed in a meta-analysis that increased levels of PA have significant effects on depression, anxiety, psychological distress, and emotional disturbance in children. Thus, regular PA seems to ensure or improve the mental health status of children and youth. The mechanisms by which this happens are multifactorial. Research proposed that PA may trigger various neurobiological mechanisms such as releasing endogenous opioid peptides in blood, as well as psychosocial changes in terms of self-perception and self-efficacy. Moreover, behavioral aspects such as the volume and quality of sleep may play a role in this context [30,31].

Thus, the purpose of this study was two-fold: first, to measure objectively the PA behavior of young people in Iran during the lockdown phase of the COVID-19 pandemic; and second, to investigate whether PA predicts the mental health indicators. We hypothesized that the majority of the Iranian adolescents do not meet the WHO guideline of 60 min MVPA per day. Furthermore, we assumed that MVPA and the incidence of mental health disorders are negatively associated and that adolescents with moderate daily MVPA report lower values of depression, anxiety, and stress than adolescents with low daily MVPA.

## 2. Materials and Methods

### 2.1. Participants

A total of 154 students aged 15 to 17 years from regular high schools of Aliabad Katoul city, Iran, voluntarily participated in this study. Of these students, 136, with an average age of 16.28 years (SD = 0.97), wore the accelerometer for at least four of the seven days for eight hours per day and thus were included in the analyses. There were 60 (44%) male students and 76 (56%) female students within the sample. The study was conducted in accordance with the declaration of Helsinki, and the University Ethics Committee approved the research protocol. Parents and adolescents were informed about all study procedures and parents provided written consent.

### 2.2. Measures

#### 2.2.1. Physical Activity

PA was measured using the accelerometer ActiGraph wGT3X-BT (ActiGraph LLC, Pensacola, FL, USA) initialized at a 30 Hz frequency. The ActiGraph wGT3X-BT captures and records high resolution human activity information using a 3-axis accelerometer. ActiGraph’s Bluetooth^®^ Smart wGT3X-BT wireless activity monitor, in conjunction with the ActiLife software platform (i.e., ActiLife v6.13.4) provides objective 24-h PA and sedentary behavior including frequency, intensity, and duration of PA, sedentary time, raw acceleration, energy expenditure, MET rates, and steps taken. Accelerometers are small (i.e., 4.6 × 3.3 × 1.5 cm), light (i.e., 19 g), non-invasive, and easy-to-wear devices that can be located on the wrist, waist, ankle, and hip. The ActiLife v6.13.4 software platform is employed to prepare ActiGraph devices for data collection and to download, process, score and securely manage collected data. In recent years, the ActiGraph accelerometer was the most frequently used in research and has consistently shown good validity and reliability in many studies [32].

#### 2.2.2. Mental Health

Mental health was measured with the Depression, Anxiety, Stress Scale-21 (DASS-21) [33]. The DASS-21 is a self-report instrument designed to assess the negative emotional states of depression (e.g., “I couldn’t seem to experience any positive feeling at all”), anxiety (e.g., “I was worried about situations in which I might panic and make a fool of myself”), and stress (e.g., “I was intolerant of anything that kept me from getting on with what I was doing”), each with seven items which refer to the past week. Answers are provided on a 4-point Likert scale ranging from “Did not apply to me at all” to “Applied to me very much, or most of the time”. Responses were summed to create a total score for each subscale (0–21), with higher scores indicating higher levels of symptoms. The psychometric properties of the DASS-21 were confirmed in adolescents [34], including the Persian version [35].

### 2.3. Procedure

Prior to implementation of the research protocol, an informational session was held online for any individual participant. In this session, information was given on the study’s aims and procedure. For collecting the data, one accelerometer was initialized, given and explained to each participant one day prior to the start of his or her individual protocol. The participants received detailed information about the accelerometer and were instructed to wear it on the right hip for seven consecutive days while awake and to remove it only for taking a shower, water-based activities, and while sleeping. They were also given an online protocol to record when and why they did not wear the accelerometer and the time of waking up and going to bed. In order to enhance the commitment of the participants and to ensure the correct use of the accelerometer, the participants were regularly contacted via WhatsApp. After seven or eight days, the accelerometers were collected from the participants. In compliance with the COVID-19 rules, each accelerometer was disinfected before and after using it. For the same reason, the DASS-21 was provided to the participants solely online via Google Forms. The data collection took place from October 2020 to February 2021. During this time, social distancing policies were adopted in Iran and schools were closed. The present study is part of the research project PAIR (Physical Activity of Children and Adolescents in Iran) which is a joint research project of Islamic Azad University (Iran) and the University of Luxembourg (Luxembourg). There were 20 accelerometers used in this study for data collection. The accelerometers are the property of the University of Luxembourg and were purchased with a research grant belonging to the last author.

### 2.4. Data Analysis

The accelerometer data were downloaded, processed, and analyzed using the ActiLife v6.13.4 software (Actigraph Inc, Pensacola, Florida, USA). Based on the cutoff points given by Evenson, Catellier, Gill, Ondrak, and McMurray [36], the total and daily time of MVPA and being sedentary were calculated. Time spent not wearing the accelerometer was identified by the algorithm by Choi, Liu, Matthews, and Buchowski [37], and subsequently matched with the information provided by the participants. The data were then analyzed with SPSS Statistics 26. Means and standard deviations were calculated to describe the MVPA data. Independent *t*-tests were computed to identify differences in MVPA and mental health between male and female students. Additionally, we conducted linear regressions to determine the amount of which daily MVPA, as well as age, gender, and BMI predict mental health status. Finally, we divided the sample into two groups according to the amount of daily MVPA. Participants with less than 20 min of daily MVPA were assigned to the low daily MVPA group and participants with more than 20 min formed the moderate daily MVPA group. Independent *t*-tests were then used to examine whether the students in these groups differ in terms of their mental health status. The level of significance was set at *p* < 0.05.

## 3. Results

### 3.1. Physical Activity

Table 1 shows the mean and standard deviation of body-related and PA data of the participants, as well as the results of the independent *t*-tests comparing boys and girls. The accelerometer data reveal that students spent 71.98% of the total time they wore the device in sedentary behavior, with no significant difference between boys and girls (*p* = 0.428). In addition, 23.19% of the total time was spent in light PA, again with no significant gender difference (*p* = 0.220). Total time of moderate PA was 3.34% and, here, male adolescents showed significantly more activity than their female peers (*p* < 0.001). Finally, only 1.49% of the total time was identified as vigorous PA; as expected, boys were on this level significantly more active than girls (*p* < 0.001). On average, the daily time spent in MVPA was 18.47 min (or 4.83% of total time), which is clearly below the WHO guideline of at least 60 min of MVPA per day. In fact, only 4% of the students in this study fulfilled the guideline. Adolescent boys engaged significantly more in MVPA per day than their female peers (*p* = 0.015). Accordingly, they also expended significantly more energy per day than the girls (*p* < 0.001).

### 3.2. Physical Activity and Mental Health

Table 2 presents the linear regression analyses with daily MVPA, age, gender, and BMI as predictor variables, and the mental health indicators, depression, anxiety, and stress, as dependent variables. The analyses show that MVPA per day is negatively associated with all of the mental health indicators; depression (F_1,134_ = 12.288, *p* = 0.001, R^2^ = 0.084, β = −0.290), anxiety (F_1,134_ = 26.069, *p* < 0.001, R^2^ = 0.163, β = −0.404), and stress (F_1,134_ = 13.096, *p* < 0.001, R^2^ = 0.089, β = −0.298). Thus, higher levels of MVPA per day significantly predict lower levels of depression, anxiety, and stress among adolescents. In contrast, neither age and gender, nor BMI were significant predictors of the mental health status (all *p* > 0.05).

**Figure 1:**
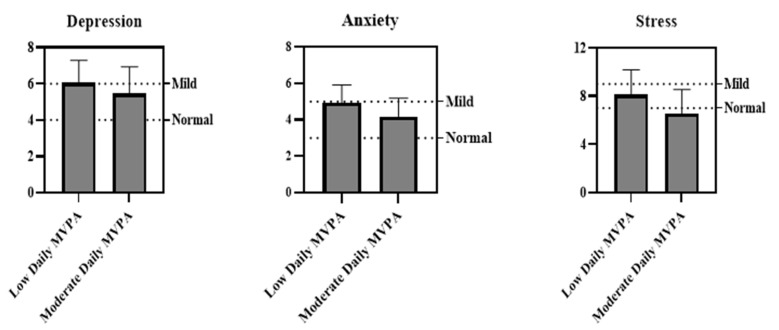
Means of depression, anxiety, and stress across different levels of daily MVPA.

Table 3 shows the mean and standard deviation of mental health data of the participants and gender differences calculated by independent *t*-tests. The results showed that 18% of the adolescents reported normal depression symptoms, 52% mild depression symptoms and 30% moderate depression symptoms. Students with moderate daily MVPA had significantly lower symptoms of depression than those with low daily MVPA (t = 2.439, *p* = 0.016) (see Figure 1). In addition, 10% of the participants perceived no anxiety, 31% mild anxiety, and 59% moderate levels of anxiety. Again, students with moderate daily MVPA had significantly lower symptoms of anxiety than those with low daily MVPA (t = 4.124, *p* < 0.001) (see Figure 1). Finally, 43% of the adolescents indicated normal stress, and 36% and 21% indicated mild or moderate symptoms of stress, respectively. As hypothesized, the analysis confirmed that students with moderate daily MVPA had significantly lower symptoms of stress than those with low daily MVPA (t = 4.112, *p* = 0.001) (see Figure 1). No gender differences were observed in all the mental health disorders (all *p* > 0.05).

## 4. Discussion

Using self-report questionnaires, several studies have reported that social isolation during the COVID-19 quarantine seriously changed the level of PA in children and adolescents [15,16,17]. Due to the limitations of self-report methods, there is uncertainty regarding the actual PA levels of children and adolescents during the pandemic. Therefore, the primary purpose of this study was to measure, for the first time, the PA of Iranian adolescents objectively with the use of accelerometers, and to determine the extent to which the PA guideline of the WHO has been met under the pandemic conditions. Moreover, we aimed to investigate whether accelerometer-measured PA is able to predict mental health indicators, namely depression, anxiety, and stress, of adolescents during the pandemic.

Concerning PA, findings revealed that students spent 71.98% of the total time in sedentary behavior, 23.19% in light PA, 3.34% in moderate PA, and only 1.49% in vigorous PA. On average, the daily time spent in MVPA was 18.47 min per day (or 4.82% of the total time), which is below the WHO guideline of at least 60 min of MVPA per day. Results also demonstrated that no adolescent accumulated the recommended 60 min or more of MVPA per day during the COVID-19 pandemic. We found that only 4% of adolescents engaged in MVPA for more than 30 min per day, and 96% of them engaged in less than 30 min of MVPA per day. This indicates how strong the impact of pandemic-related restrictions on PA among adolescents actually was, and it is certainly alarming. Although numerous studies have examined the PA behavior of children and adolescents during the pandemic, only a few of them have reported exact MVPA per minute data, probably due to the limitations of self-report methods. Morres et al. [38] found that adolescents in Greece on average spent only 23.9 min per day in moderate-to-vigorous physical activity, while Tulchin-Francis et al. [39] revealed for children and adolescents in the United States an amount of 34.7 MVPA minutes per day. In both studies, questionnaires were used to assess PA; however, the findings are similar to our results and demonstrate that the PA of children and youth in many countries clearly declined under the 60 min recommendation of the WHO. This is also shown by studies in which the PA level of children and adolescents before and during the pandemic was compared [15,17,40]. Overall, research in this field has revealed to a worrying extent that the COVID-19 pandemic has seriously and negatively affected the PA patterns in children and adolescents.

Concerning gender-related differences in PA, we found that adolescent boys engaged significantly more in moderate PA, vigorous PA, total MVPA, and MVPA per day, as well as expending significantly more energy than their female peers. This is in accordance with the research in this field [41,42,43,44,45]; however, current studies show inconsistent results for gender differences in PA during the pandemic. For instance, Tulchin-Francis et al. [39] found no significant differences in PA behavior between male and female students, while Moore et al. [40], as well as Morres et al. [38], reported significant gender differences in PA and sedentary behaviors, with boys scoring higher in PA and lower on sedentary time. As mentioned above, we also found that boys were significantly more physically active than girls during the pandemic or the lockdown. Given the fact that schools were closed at this time and both male and female students had no physical education or other school-related opportunities for PA, these discrepancies may be due other reasons. Sport clubs, municipalities and even the parents possibly offered more (or more appropriate) activities for boys than for girls during the lockdown. Furthermore, reliable evidence exists that male and female adolescents differ in their motivation for being physically active, because they receive gender-specific socialization in terms of sports and movement [46,47,48]. Future research should focus on identifying factors influencing gender differences in PA in school-students, particularly during the COVID-19 pandemic, bearing in mind that these factors may differ among cultures and countries.

Concerning mental health, results showed that 82% of the students reported symptoms of mild-to-moderate depression, 90% had symptoms of mild-to-moderate anxiety, and 57% had symptoms of mild-to-moderate stress during the pandemic. No differences were found between male and female students regarding mental health. These findings are in line with those of previous studies showing that the COVID-19-related containment measures have a detrimental impact on mental health of children and adolescents in different countries across the globe [23,24,49,50]. This is quite understandable due to many changes in the daily lifestyle of children and adolescents during the quarantine. The lack of opportunities for regular social contacts with peers, loneliness, fear of illness or death of relatives, problems related to health services, financial loss, etc. are factors that potentially cause or reinforce mental health disorders such as depression and anxiety [24,49,51]. However, two important findings in the present study indicate that PA plays an important role in reducing the symptoms of mental health disorders. First, we compared mental health (i.e., depression, anxiety, and stress) of adolescents with low or moderate daily MVPA. Interestingly, our results showed that adolescents with moderate daily MVPA had significantly lower levels of mental health disorders than those with low daily MVPA. Moreover, the results of linear regression analysis revealed that MVPA per day is in fact a significant predictor for depression, anxiety, and stress. These findings are in line with those of previous studies [26,27,28,29,47,51,52] and indicate that engaging in PA can be considered as an important resource for coping with mental health disorders among adolescents and possibly other age groups during the COIVD-19 pandemic. As mentioned earlier, the mechanisms underpinning the effects of PA on mental health are multifactorial and consisted of releasing endogenous opioid peptides in the blood, enhancing physical self-perceptions and self-efficacy, and better sleep volume and quality [30,31]. Future research should aim to focus on examining MVPA interventions based on self-perceptions and self-efficacy to determine whether they are mechanisms for improving students’ mental health in a real-world setting (e.g., school) or during the pandemic-related social isolation.

The strengths of our study are, firstly, the use of up-to-date accelerometers to determine objectively the amount and levels of PA and sedentary behavior of adolescent students during the COVID-19 pandemic. In this way, it was possible to prevent typical biases which are often associated with self-reporting methods. Secondly, we measured PA and mental health of both male and female adolescents which made it possible to examine gender differences. However, our study is limited in the sense that we did not include variables which might also be of importance regarding the relationship of PA and mental health, such as the socio-economic status of the family, the local environment (e.g., availability of sports facilities near to the residence), and individual factors (e.g., motivation, attitudes, physical self-concept). Further research should consider some of these variables in order to get a more comprehensive insight into how PA of adolescents is embedded in their individual and socio-economic environment. Due to restrictions related to the COVID-19 pandemic and the high demands of accelerometer studies in terms of temporal organization and participant’s commitment, the sample of the present study was relatively small compared to studies which were realized prior to the pandemic or using questionnaires.

## 5. Conclusions

This study was one of the first to objectively measure the PA of adolescents during the COVID-19 pandemic. Our findings showed that our cohort did not accumulate the recommended 60 min or more of MVPA per day during the lockdown phase of the pandemic. Indeed, on average, the daily time spent in MVPA was only 18.47 min per day. At this low level, boys engaged significantly more in MVPA than their female peers. Moreover, many adolescents reported symptoms of mild-to-moderate mental health disorders. Altogether, these findings indicate that PA and mental health have been and still are critical issues for adolescents during the COVID-19 pandemic. Interestingly, our findings showed that adolescents with at least a moderate amount of MVPA have a better mental health than their fewer active peers. Apparently, PA acts as a resource that prevents or at least relieves symptoms of mental health disorders such as depression and anxiety. In future lockdown periods it should therefore be ensured that children and adolescents still have access to sport and exercise, for example, by keeping sports clubs and facilities open in compliance with current COVID-19 regulations.

## Figures and Tables

**Table 1:** Body-related and physical activity data of the participants.

Variables	Overall		Boys (*n* = 60)		Girls (*n* = 76)		Results of *t*-Tests
	Mean	SD	Mean	SD	Mean	SD	
Age	16.28	0.97	16.35	0.98	16.23	0.96	t = 0.672 *p* = 0.503
Height (cm)	164.97	10.29	165.23	10.68	164.77	10.03	t = 0.256 *p* = 0.798
Weight (kg)	63.25	10.03	64.36	10.47	62.36	9.64	t = 1.155 *p* = 0.250
BMI (kg/m^2^)	23.28	3.49	23.63	3.71	23.00	3.31	t = 1.044 *p* = 0.298
**Accelerometer Data**							
% Sedentary Behavior	71.98	3.13	71.80	1.53	72.38	3.95	t = 0.794 *p* = 0.428
s% Light PA	23.19	2.24	22.83	0.88	23.30	2.89	t = −0.713 *p* = 0.220
% Moderate PA	3.34	0.90	3.59	0.92	3.05	0.64	t = −3.568 *** p = 0.000
% Vigorous PA	1.49	1.06	1.78	0.88	1.27	1.14	t = 4.005 *** p = 0.000
% MVPA	4.83	1.57	5.37	1.66	4.32	1.45	t = −4.651 *** p = 0.000
Total MVPA (min)	135.68	44.58	144.87	43.38	124.05	36.40	t = −2.771 ** p = 0.006
Daily MVPA (min)	18.47	6.46	19.66	7.10	16.96	5.22	t = −2.461 * p = 0.015
Daily Energy Expenditure (Kcal)	306.63	76.26	2368.32	608.97	2131.92	514.23	t = −2.405 * p = 0.018

* *p* < 0.05; ** *p* < 0.01; *** *p* < 0.001; PA: physical activity; MVPA: moderate-to-vigorous physical activity; BMI: body mass index.

**Table 2:** Prediction of mental health from MVPA, age, gender, and BMI.

	Depression	Anxiety	Stress
MVPA	β = −0.290 t = −3.505 R^2^ = 0.084 F_1,134_ = 12.288 *	β = −0.404 t = −5.106 R^2^ = 0.163 F_1,134_ = 26.069 **	β = −0.298 t = −3.619 R^2^ = 0.089 F_1,134_ = 13.096 **
Age	β = −0.142 t = −1.660 R^2^ = 0.020 F_1,134_ = 2.755	β = −0.123 t = −1.450 R^2^ = 0.018 F_1,134_ = 2.325	β = −0.113 t = −1.317 R^2^ = 0.013 F_1,134_ = 1.735
Gender	β = −0.033 t = −0.380 R^2^ = 0.001 F_1,134_ = 0.144	β = 0.084 t = 0.979 R^2^ = 0.007 F_1,134_ = 0.959	β = 0.117 t = 1.368 R^2^ = 0.014 F_1,134_ = 1.871
BMI	β = −0.071 t = −0.819 R^2^ = 0.005 F_1,134_ = 0.670	β = −0.090 t = −1.041 R^2^ = 0.008 F_1,134_ = 1.084	β = −0.146 t = −1.712 R^2^ = 0.021 F_1,134_ = 2.932

* *p* < 0.01; ** *p* < 0.001; MVPA: moderate-to-vigorous physical activity; BMI: body mass index.

**Table 3:** Mental health data of the participants.

Mental Health	Overall		Boys (*n* = 60)		Girls (*n* = 76)		Results of *t*-Tests
	Mean	SD	Mean	SD	Mean	SD	
Depression	5.86	1.33	5.91	1.36	5.82	1.31	t = 0.380 *p* = 0.705
Anxiety	4.69	1.04	4.60	1.02	4.77	1.05	t = −0.979 *p* = 0.329
Stress	7.61	2.16	7.33	2.28	7.84	2.04	t = −1.368 *p* = 0.174

## Data Availability

The data presented in this study are not publicly available due to ethical reasons.
